# Ultrafast Microwave Nano-manufacturing of Fullerene-Like Metal Chalcogenides

**DOI:** 10.1038/srep22503

**Published:** 2016-03-02

**Authors:** Zhen Liu, Lin Zhang, Ruigang Wang, Selcuk Poyraz, Jonathan Cook, Michael J. Bozack, Siddhartha Das, Xinyu Zhang, Liangbing Hu

**Affiliations:** 1 Department of Chemical Engineering, Auburn University, Auburn, AL 36849, USA; 2 Department of Materials Science and Engineering, University of Maryland, College Park, MD 20742-4111, USA; 3 Materials Research and Education Center, Auburn University, Auburn, AL 36849, USA; 4 Department of Chemistry, Youngstown State University, Youngstown, OH 44555, USA; 5 Surface Science Laboratory, Department of Physics, Auburn University, Auburn, AL 36849, USA; 6 Department of Mechanical Engineering, University of Maryland, College Park, MD 20742-4111, USA

## Abstract

Metal Chalcogenides (MCs) have emerged as an extremely important class of nanomaterials with applications ranging from lubrication to energy storage devices. Here we report our discovery of a universal, ultrafast (60 seconds), energy-efficient, and facile technique of synthesizing MC nanoparticles and nanostructures, using microwave-assisted heating. A suitable combination of chemicals was selected for reactions on Polypyrrole nanofibers (PPy-NF) in presence of microwave irradiation. The PPy-NF serves as the conducting medium to absorb microwave energy to heat the chemicals that provide the metal and the chalcogenide constituents separately. The MCs are formed as nanoparticles that eventually undergo a size-dependent, multi-stage aggregation process to yield different kinds of MC nanostructures. Most importantly, this is a *single-step* metal chalcogenide formation process that is much faster and much more energy-efficient than all the other existing methods and can be universally employed to produce different kinds of MCs (e.g., MoS_2_, and WS_2_).

During the past decades, intense interests have been aroused in metal chalcogenides because of their unique properties and promising applications^1^,^2^,^3^,^4^. The first closed-cage inorganic fullerene-like nanoparticles (IFs), layered MoS_2_ and WS_2,_ were reported around 1990’s^1^,^2^,^3^. After a decade-long effort, many other layered compounds, such as transition metal chalcogenides MX_2_ (M = W, Mo, Sn, Ti, Re, Nb, Ta, Hf and Zr, etc, X = S, Se)^5^, are now achievable. Recently, MX_2_ has been investigated extensively because of their unique layered structures and excellent physical and chemical properties, which make them ideal candidates for use as lubricants^6^,^7^,^8^,^9^, heterogeneous catalysts^10^,^11^,^12^, solar cells^13^, lithium-ion batteries^14^,^15^, hydrogen storage elements^16^,^17^ and high performance protective composites^18^,^19^,^20^.

There has been significant progress made in synthetic methods for MS_2_ production, characterization, and in investigation of its physical and chemical properties as well as various applications. Synthetic methods for MS_2_ production mainly consist of (1) *chemical methods* such as solid-gas or gas-phase reactions^3^,^21^, thermal decomposition^22^,^23^, hydrothermal or solvothermal synthesis^24^,^25^, and template synthesis^26^ and (2) *other instant stimulation methods* such as laser ablation^27^, arc discharge^7^,^28^, and electron beam irradiation^29^,^30^. Despite major progress, there are still several limitations and disadvantages associated with the existing MS_2_ fabrication techniques. Some of them include the presence of extreme reaction conditions (high temperature, argon protection), involvement of many toxic and hazardous gases (e.g. H_2_S) and complicated processes, requirement of intense facilities (e.g., furnace, laser, arc discharge, and high voltage beams), and many more^31^,^32^,^33^,^34^,^35^. These challenges warrant the need to develop a method that can synthesize high-purity but low cost products of MS_2_ at industrial scales.

Very recently, there has been a major surge in employing microwave-based ultrafast, energy-efficient, and facile approaches for synthesizing multi-component nanostructures (e.g. metal oxides, metal sulfides)^36^,^37^,^38^,^39^,^40^,^41^,^42^,^43^. There are still some limitations in previous reports of microwave-assisted preparations for metal oxides/sulfides. Firstly, the effective microwave-assisted strategy has been developed for assisting other synthesis methods, such as solvothermal approach, which means the microwave heating is not the only or main tool during products preparation^36^,^37^. Secondly, the microwave irradiation has to treat the sample for relative long time (more than 30 min or several hours)^38^,^39^. Lastly, the selection of useful and effective precursor is the key to uniform growth of metal oxides/sulfides. The directly mixture of Mo (or W) with S could not lead to the formation of MoS_2_ (or WS_2_) by microwave heating^41^. Synthesis of precursor involved multi-step processing in some studies, which were not the simple and cost-effective processing way^42^.

In this communication, we report our discovery of a facile and energy-efficient route with multi-scale aggregation dynamics for the microwave-assisted synthesis of IF-MS_2_ nanoparticles. Our technique represents a clean, ultrafast (60 seconds) nano-manufacturing approach using microwave heating without any inert gas protection and intense facilities^44^,^45^,^46^,^47^. Figure 1 provides the schematic of our hypothesized mechanism of the process. We start with a uniform blend of polypyrrole nanofibers (PPy-NF) and M(CO)_6_ (M = Mo, W) forming homogenous dark powder composites. This blend is mixed with sulfur (S) powder or S/CS_2_ solution, and is subjected to microwave irradiation (frequency 2.45 GHz, power 1250 W MW) for 60 seconds. Polypyrrole nanofibers has been synthesized using our previously developed seeding polymerization methods^48^,^49^ and serves as a substrate owing to its low cost, relatively high electrical conductivity, tunable doping/de-doping characteristics, and long-term environmental stability. Microwave heating triggers reaction between M(CO)_6_ and sulfur or S/CS_2_ solution. This reaction first leads to the formation of MS_2_ nanoparticles, with M(CO)_6_ providing the metal element. These nanoparticles undergo intense heating which promotes molecular collisions and the formation of aggregates. In this first stage of aggregation, where the nanoparticle sizes being too small (~few nms), van der Waals (vdW) interactions govern the aggregation of particles leading to spherical growth of the aggregate. The second stage of aggregation involves nanoparticles with much larger diameters (~100 nms) and the difference in respective surface energies (nanoparticle-air and nanoparticle-nanoparticle) becomes dominant to lead to directional growth of the aggregate. This results in a ring-like growth of MoS_2_ aggregates (see Fig. 1), but a spherical growth of MoO_x_ aggregates (not depicted in Fig. 1), which is the product of microwave heating the mixture of PPy-NF and M(CO)_6_.

## Results

### Synthesis of MoO_x_ nanoparticles by microwave approach

The proposed microwave-based nano-manufacturing set up is first calibrated by producing MoO_x_ nanoparticles (the corresponding figures are in the Supplementary Information or SI). Figure S1 shows the SEM images of MoO_x_ particles decorated on the PPy nanofibers, using the microwave reaction with Mo(CO)_6_ precursor. The MoO_x_ particles uniformly distribute on the PPy network. On a given PPy-NF, the MoO_x_ particles fully cover the surface of fiber. The PPy-NF serves to conduct the microwave heat to Mo(CO)_6_, yielding MoO_x_. The inserted EDS spectrum is used to confirm the particles are mainly composed of molybdenum and oxygen and the presence of gold (Au) is due to the gold coating for SEM imaging. The XPS result in Figure S2 shows the ratio of molybdenum and oxygen. The spectra are similar to the literature reference XPS spectra for pure polypyrrole^50^, with the addition of small amount of Si, which is related to a surface contaminant and/or associated with the deposition method. The signals from O1s are larger than the reference spectrum, while that from the N1s are smaller. The literature reports the polypyrrole surface composition as: C: 72%, O: 14%, N: 11%. The polypyrrole investigated here is: C: 75%, O: 21%, N: 2%. The larger signal of O is due to the presence of the MoO_x_ particles and adventitious O due to atmospheric exposure. The Mo3d5/2 BE (Bond Energy) is close to MoO_3_ and/or native MoO_2_. Figure S3 shows the X-ray diffraction (XRD) patterns of MoO_x_ particles. According to MoO_3_ (JCPDS No. 05-0508), the diffraction peaks at following angle, such as 14°, 33°, 39°, and 58° correspond to the (020), (111), (060), and (171) planes of the MoO_3_, respectively. According to MoO_2_ (JCPDS No.86-0135), for example, the merged peaks at 37° and 54° are attributed to the 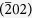,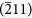, (200) and 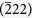, 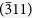 (220) planes of the MoO_2_, respectively. The *x* in MoO_x_ cannot be exactly confirmed which has a mixed Mo^4+^ and Mo^6+^ valence states. This is similar to a previous report in synthesis of mixed-valence MoO_x_ on carbon nanotube^51^ and synthesis of WO_x_ from W(CO)_6_^33^.

### Synthesis of IF-MS_2_ nanoparticles by microwave heating

Using the same conditions and process, MoS_2_ particles can be easily produced by only adding sulfur powder to the initial mixture. Figure 2(a,b) shows the SEM images of MoS_2_ particles on the “bed” of PPy-NF. The MoS_2_ particles are aggregated together on PPy-NF. The EDS result shown in Fig. 2(c) indicates that particles are primarily composed of molybdenum and sulfur. The XPS results in Figure S4 indicates the Mo3d5/2 BE (Bond Energy) aligns favorably with both MoS_2_ and MoO_2_ and the S2p BE aligns favorably with the BE for a standard XPS spectrum of MoS_2_. The XRD patterns of the MoS_2_ display diffraction peaks in the range from 10° to 80°, as shown in Fig. 2(d). The patterns can be indexed to the standard hexagonal 2H–MoS_2_ structure (JPCDS no. 37-1492), which indicates the MoO_x_ composites with different ratio of MoO_2_/MoO_3_ will completely become MoS_2_. Advanced TEM has been widely utilized as a very powerful instrument for the analysis of nanoparticle synthesis in recent studies to determine the crystal structure and for surface reconstruction in both particles and thin films^52^,^53^,^54^. Figure 3(a) shows the typical assortment of IF-MoS_2_ nanoparticles obtained directly by microwave heating. A closer look at these nanoparticles (Fig. 3(b,c)) reveals the particles are hexagonal or heptagonal in shape and possess a compact multiwall structure (>25 walls) with a prominent oxide nanoparticle core, as shown in Fig. 3(b). The line profile of the framed area (box 1) indicates that the spacing distance of the interlayer is around 0.62 nm (6.172 nm of 10 layers) [see Fig. 3(d)], which is very close to the separation of the (002) planes of MoS_2_ (c/2 lattice spacing). The hexagonal atomic arrangement shown Fig. 3(f) and the FFT pattern indicate that the basal plane of the synthesized thin film is (001), i.e. the c-axis of MoS_2_ materials perpendicular to the thin film. Each sulfur center is pyramidal and is connected to three Mo centers. The lattice constant is a = b = 0.315 nm. Along the wall, the distance between the Mo is 
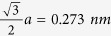
 which is very close to the measured value of 0.261 nm [see Fig. 3(e)]. The results discussed above show that the microwave nano-manufacturing can easily prepare the inorganic fullerene-like MoS_2_ in a single step. To confirm its robustness, we employ this method to prepare other metal chalcogenides (e.g., IF-WS_2_ from W(CO)_6_ and sulfur source). The TEM images of IF-WS_2_ thus prepared are shown in Figure S5. The size of both IF-WS_2_ and IF-MoS_2_ metal chalcogenides is similar, but the shape of IF-WS_2_ nanoparticles is more spherical. The size of metal-oxide core is much smaller than the core in IF-MoS_2_ nanoparticles. The multiwall structure is not well compacted and well-organized, and the number of layers can be more than 40. The interlayer spacing is around 0.613 nm and the distance between layers of W in one plane is around 0.220 nm, both of the values are smaller than the value of IF-MoS_2_ nanoparticles.

### Mechanism of IF-MS_2_ formation

**Figure 1:**
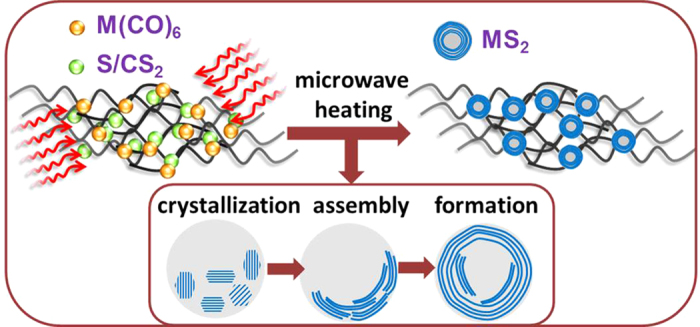
Schematic of the experimental process.

**Figure 2:**
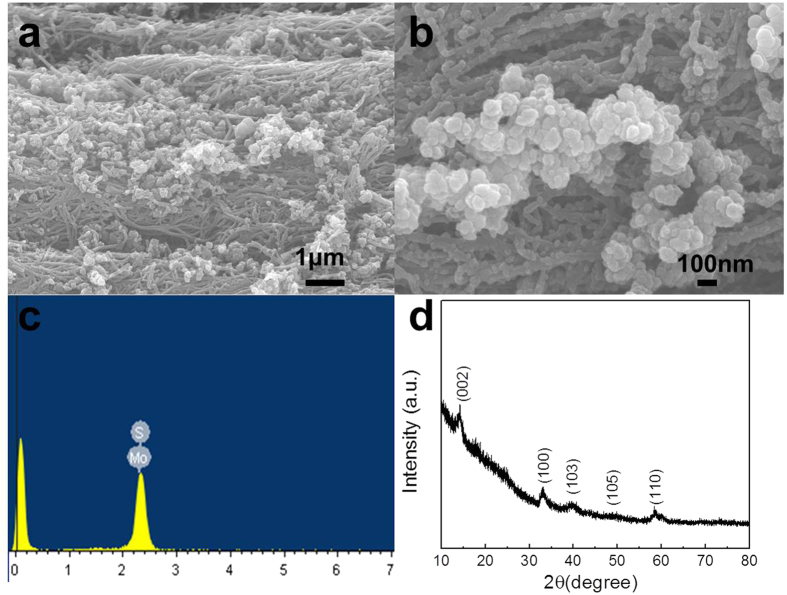
SEM images (a, b) of PPy fiber and MoS_2_ particles, EDS analysis and XRD pattern (c, d) of MoS_2_ particles.

**Figure 3:**
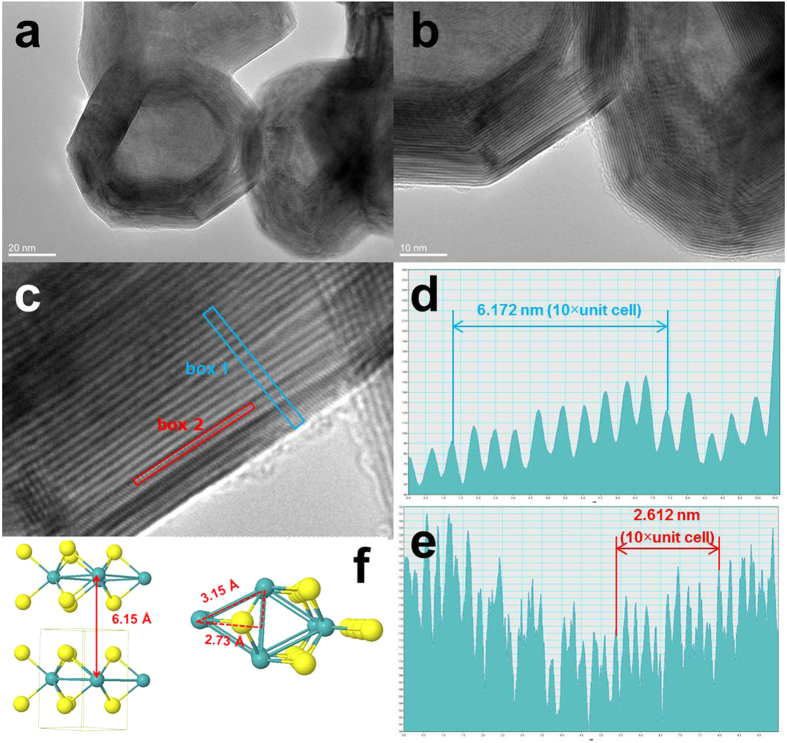
(**a**) and (**b**) HRTEM images of MoS_2_ particles, (**d**) and (**e**) line profiles of the framed area in (**c**), (**f**) The schematic of MoS_2_ structure.

**Figure 4:**
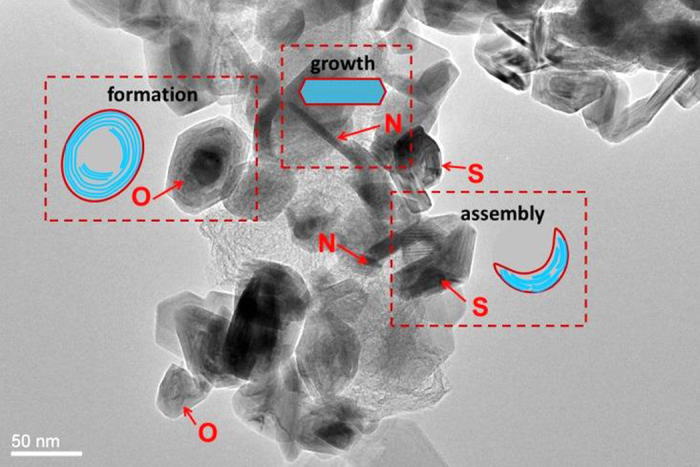
HRTEM image of MoS_2_ particles: (N) needle-like, (O) onion-like and (S) irregular shaped particles.

**Figure 5:**
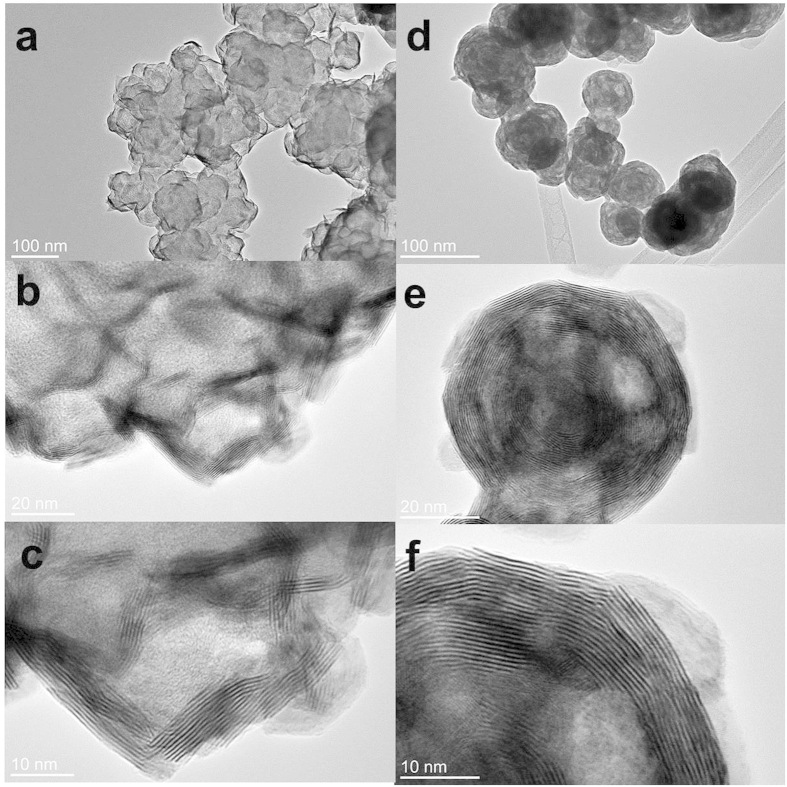
HRTEM images of WS_2_ particles: (**a–c**) growth and assembly, (**d–f**) formation.

To the best of our knowledge in all the existing studies, the synthesis of inorganic fullerene-like MS_2_ is performed as a two-step process, with the synthesis of nanoparticles being followed by annealing in inert atmosphere in a conventional tube furnace^4^,^55^. In this novel microwave approach, the temperature generated by microwave heating can reach above 1000 °C for synthesizing MoS_2_ directly^56^. The reaction is similar to the MOCVD which involves the reaction between organic gas and metal particles at very high temperature (>700 °C)^55^. The schematic experimental procedure is shown in Fig. 1. The particles of M(CO)_6_ and particles of sulfur contact well with each other on the networks of PPy. The PPy networks provide the channel of conduction and pathway of heating. When microwave energy is applied, the reactions occur and the amorphous nuclei are formed. Tangential MS_2_ layers are gradually formed from the edges. As shown in Fig. 4, there are many different shapes and intermediates of MoS_2_, such as needle-like (N), onion-like (O), and irregular (S) shaped particles, which is consistent to the prior report, that uses MOCVD technique and post-steps (i.e., annealing under argon protection)^56^,^57^. Comparing to this MOCVD method, the microwave approach can directly synthesize these segments of multi-layers in different stages of growth of MoS_2_, such as crystallization, assembly and formation discussed in Fig. 1. The presence of this mechanism can also be proved in growth of WS_2_. Figure 5 clearly shows the intermediates of WS_2_ in the process of assembly. Very thin layers (3–5 layers) are assembled from the edges of amorphous particles and form irregular boundaries as shown in Fig. 5(a–c). The crystallization process was shown to start at the outer surface of the round nanoparticle and propagate inwards. High temperature or thermal stress may initiate bending or faceting of the crystals. The strong inter-layer covalent bonding causes the crystal (layers) to close by itself and the structure is expected to be stable, as observed in Fig. 5(d–f). Finally, closed-cage inorganic fullerene-like nanoparticles of WS_2_ are formed. It should be mentioned that, particles in Fig. 5(a) are all in stages of growth and assembly while the particles in Fig. 5(d) exhibit the IF morphology. It indicates that these particles undergo uniform microwave heating and concurrent morphological change.

Recently, metal chalcogenides nanoparticles, tubes, and sheets were generated by a synthetic pathway that employs CS_2_ as the source of sulfur instead of H_2_S^4^. In this study, CS_2_ was also selected as co-precursor to obtain the metal chalcogenides. Figure S6 shows the TEM images of IF-MoS_2_ and IF-WS_2_ nanoparticles synthesized from Mo(CO)_6_ or W(CO)_6_ with S/CS_2_ solution. Compared with products obtained from pure sulfur powders, both metal chalcogenides are not well faceted. The number of layer is less than those produced by sulfur powders. These cage-like materials possess different morphologies such as perfect spheres, semispherical particles or structures in which crystalline directions were evident and resulted in polyhedral shapes. For IF-MoS_2_ in Figure S6(a–c), due to the incomplete closure of the outermost layers, hair-like outgrowths were evident in the structure. For IF-WS_2_ in Figure S6(d–f), the structure is loose and the metal-oxide core is much larger than those obtained from the reaction with sulfur powders. The results indicate that the use of S/CS_2_ solution for the generation of MS_2_ is very different from the use of S powder only. Firstly, the S powders are homogeneously dispersed in CS_2_ solvent, which can improve the uniformity of reaction between metal and sulfur. Secondly, CS_2_ vapor may serve as additional sulfur source to obtain these MCs. Thirdly, using S/CS_2_ solution may provide a potential way to synthesize MCs with few layers or even single layer.

## Discussion

**Figure 6:**
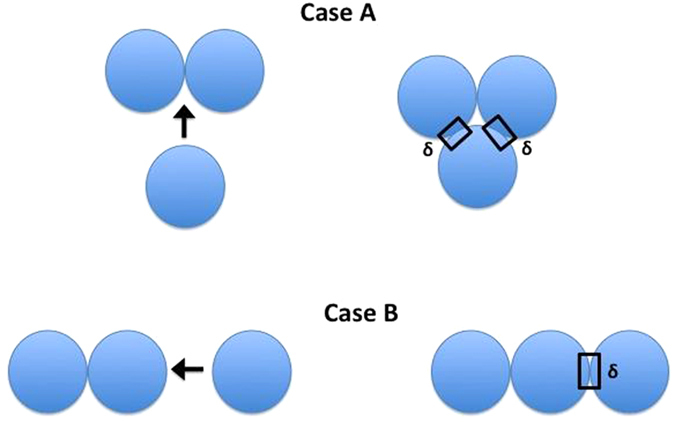
Schematic of the two possible aggregation mechanisms of large (~100 nm) nanoclusters.

Central to this single-step formation of the microwave-assisted metal chalcogenides nanostructures is the two-step aggregation process. The first stage of aggregation involves the *just-formed* MoS_2_ and MoO_x_ nanoparticles of dimensions of only few nanometers. These nanoparticles are at a thermally excited state, they vibrate rapidly and as a consequence two of these nanoparticles come to close enough proximity of each other to allow van der Waals (vdW) attractive interactions to become influential triggering the aggregation of these two nanoparticles. This aggregation displays no directional preference, as a result, the nanocluster grows in a spherical form. Things change when these nanoclusters have grown sufficiently large (~100nm). As shown in Fig. 6, when two typical spherical nanoclusters, each of 100 nm in diameter, come in close enough proximity, vdW effects dictate their possible aggregation. However, when a third nanocluster attempts to join this newly formed aggregate, two arrangements are possible, as illustrated in Fig. 6. For case A, two contact zones (each of area δ) are being created and the consequent change in the surface energy is ΔU = 2δ(γ_pp_ − γ_pa_), on the other hand for case B, only one contact zone (of area δ) is formed and the consequent change in the surface energy is ΔU = δ(γ_pp_ − γ_pa_). Here γ_pp_ and γ_pa_ are the particle-particle and particle-air surface tension values. When γ_pp_ < γ_pa_, case A occurs, since this leads to larger energy decrease (as compared to case B) due to aggregation – this typically occurs for MoO_x_. On the other hand, when γ_pp_ > γ_pa_, case B occurs, since this leads to less energy increase (as compared to case A) due to aggregation – this typically occurs for MoS_2_. Therefore, we see MoO_x_ growing as a bulk cluster [Figure S1], whereas MoS_2_ growing as a ring.

To conclude, in this communication we have reported our discovery of a single-step, universal microwave–assisted technique for producing different kinds of MC nanostructures. The process is ultrafast and extremely energy efficient. The process is based on three key steps: (a) microwave-heating assisted reaction yielding MC nanoparticles, (b) thermal vibration of these NPs to trigger aggregation and formation of larger sized nanoclusters and (c) preferential growth of the these nanoclusters, dictated by the relative preference of surface interactions forming different nanostructures. Results indicate that the morphological specifications of these nanostructures strongly depend on the nature of the MCs as well as the reactants used to obtain these MCs. We anticipate that our discovery of this new technique for preparing MCs will catalyze substantial development in more widespread uses of MCs in a more energy-efficient and easily-accessible approach.

## Methods

### Synthesis of MO_x_ nanoparticles by PPy NF and M (CO)_6_

Polypyrrole nanofibers (PPy NF) were synthesized via previously reported methods. PPy NF and Mo(CO)_6_ (or W(CO)_6_) were uniformly blended to form homogenous dark powder composites, specifically 50 mg PPy NF and 50 mg Mo(CO)_6_ (or W(CO)_6_) were blended by speed mixer at 3500 rpm for 2 min.

The aforementioned mixture of PPy NF and Mo(CO)_6_ (or W(CO)_6_) was transferred into a glass vial and loosely capped, and then placed into the microwave chamber. The solid mixture was continuously irradiated by 1250 W MW power for 60 seconds. Vigorous sparking was observed within the first 3 seconds of microwave irradiation. Particular attention ought to be given to the fast microwave reaction with extreme high temperature (approximately 1000 °C). The mixture was kept reacting (burning) for another 40 s before becoming stable in the glass vial, and showing no further signs of reaction. Eventually, after microwaving, the vials were removed and allowed to cool naturally to room temperature. The remaining solids were collected with a steel spatula and stored for further tests and characterizations.

### Synthesis of IF-MS_2_ nanoparticles by adding sulfur

A tri-component system (I) was prepared by mixing PPy NF, Mo(CO)_6_ (or W(CO)_6_) and elemental sulfur, in order to generate MoS_2_ or WS_2_ on PPy substrate. Specifically, PPy NF (50 mg), Mo(CO)_6_ (50 mg) and sulfur (50 mg) were homogenously mixed in speed mixer at 3500 rpm for 2 min. The mixtures were transferred into glass vials, loosely capped and placed into the MW chamber. The mixture was continuously irradiated by 1250 W MW power for 1 min. The reaction indicated by vigorous evolution of yellow smoke within 5 s, while taking an average of 20–30 s for these samples to start burning and glowing. The mixture continued to burn for the next 40 s, after which the sample was allowed to cool to room temperature. The as-obtained samples were collected from the glass vial bottom with a steel spatula and stored for further tests and characterizations.

### Synthesis of IF-MS_2_ nanoparticles by adding CS_2_

Another tri-component system (II) of 50 mg PPy NF, 50 mg Mo(CO)_6_ (or W(CO)_6_) and 50 mg elemental sulfur has been mixed homogeneously via speed mixer at 3500 rpm for 2 min. The mixtures were transferred into glass vials and were soaked in 2mL CS_2_ to fully dissolve the sulfur for better homogeneity and to provide more C and S into the reaction. The overall reaction was more vigorous due to the high activity of CS_2_ under heating. The mixture was then continuously irradiated by 1250 W MW power for 60 s, within 10 s the samples began reacting, indicated by the presence of bluish flames. It took an avg. of 20–30 s for samples to start burning, with glowing and evolution of bluish smoke continuing for the next 40 s. Finally the as-obtained samples were cooled and collected from the glass vial bottom with a steel spatula and stored for further tests and characterizations.

### Characterization

Scanning electron microscopy (SEM) characterization was performed on a JEOL JSM-7000F instrument. The morphology of nanoparticles was also observed by high resolution transmission electron microscopy (TEM) in JEOL 2100 microscopes at an acceleration voltage of 200 kV. The X-ray photoelectron spectroscopic (XPS) elemental analysis of the samples was conducted with a Kratos XSAM 800 instrument. The X-ray diffraction (XRD) patterns of nanoparticles were performed on a Bruker D8 Advance diffractometer.

## Additional Information

**How to cite this article**: Liu, Z. *et al*. Ultrafast Microwave Nano-manufacturing of Fullerene-Like Metal Chalcogenides. *Sci. Rep*. **6**, 22503; doi: 10.1038/srep22503 (2016).

## Supplementary Material

Supplementary Information
