# Supplementary material for: Ultrafast Microwave Nano-manufacturing of Fullerene-Like Metal Chalcogenides
Source: Sci Rep. 2016 Mar 2;6:22503. doi: 10.1038/srep22503 (PMC4773880; doi:10.1038/srep22503)

Ultrafast Microwave Nano-manufacturing of Fullerene-Like Metal Chalcogenides

Zhen Liu[1,2,†], Lin Zhang[3†], Ruigang Wang[4], Selcuk Poyraz[1], Jonathan Cook[1], Michael J. Bozack[5], Siddhartha Das[6], Xinyu Zhang[1]*, Liangbing Hu[2]*

[1]*Department of Chemical Engineering, Auburn University, Auburn, AL 36849, USA*
[2]*Department of Materials Science and Engineering, University of Maryland, College Park, MD 20742-4111, USA*
[3]*Materials Research and Education Center, Auburn University, Auburn, AL 36849, USA*
[4]*Department of Chemistry, Youngstown State University, Youngstown, OH 44555, USA*
[5]*Surface Science Laboratory, Department of Physics, Auburn University, Auburn, AL 36849, USA*
[6]*Department of Mechanical Engineering, University of Maryland, College Park, MD 20742-4111, USA*

[†] *These authors contributed equally to this work.*
**Corresponding Authors:* xzz0004@auburn.edu, binghu@umd.edu

Supporting Information

Supplementary Figures

Figure S1 (a) and (b) SEM images of MoO_x particles on PPy fibers (inset: EDS spectrum is collected on area of image (b)).

Figure S2 XPS spectrum of MoO_x particles on PPy nanofibers

Figure S3 XRD pattern of MoOx particles: solid square MoO_2 and solid triangle MoO_3.

Figure S4 XPS spectrum of MoS_2 particles on PPy nanofibers

Figure S5 (a) and (b) HRTEM images of WS_2 particles on PPy nanofibers; (d) and (e) line profiles of the framed areas (c) and (f).

Figure S6 (a), (b) and (c) TEM images of MoS_2 particles; and (d), (e) and (f) TEM images of WS_2 particles

Figure S1

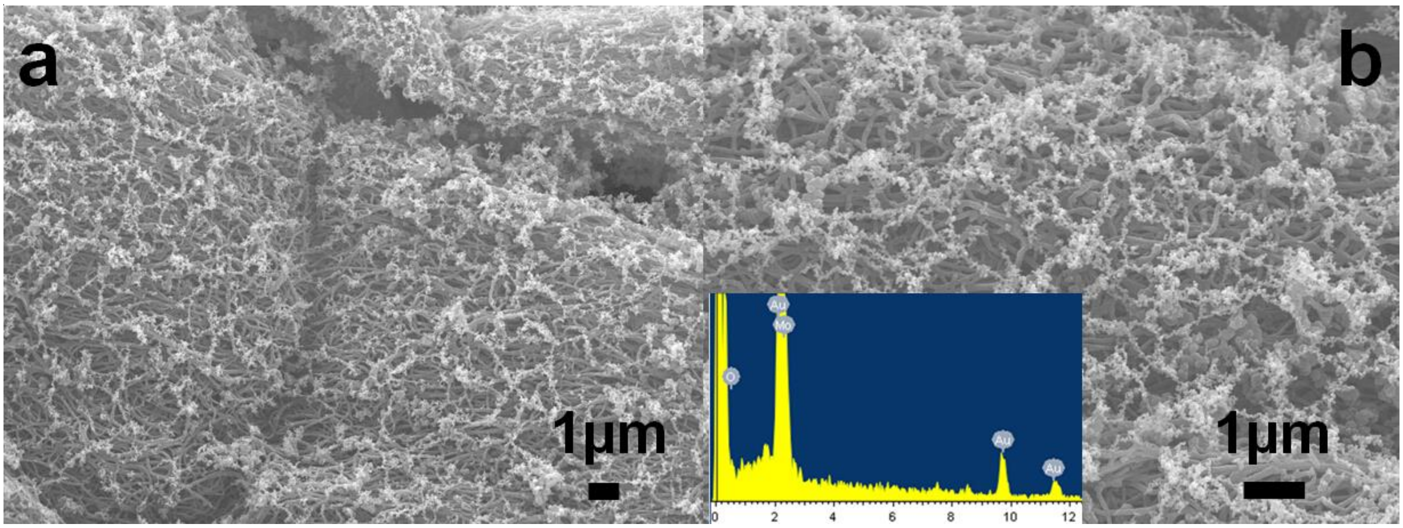

Figure S2

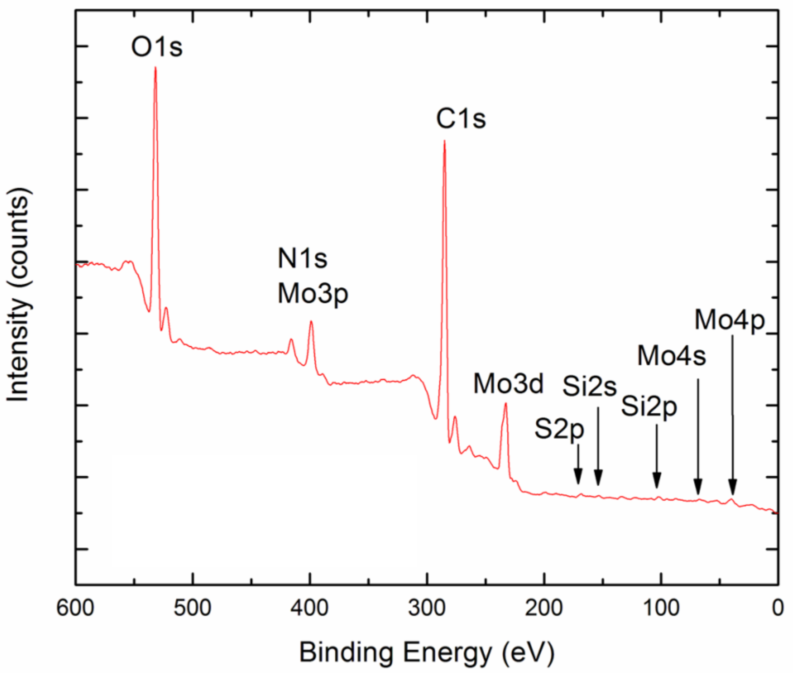
O1s
C1s
N1s
Mo3p
Mo4p
Mo4s
Mo3d
Si2s
Si2p
S2p
Intensity (counts)
600
500
400
300
200
100
0
Binding Energy (eV)

Figure S3

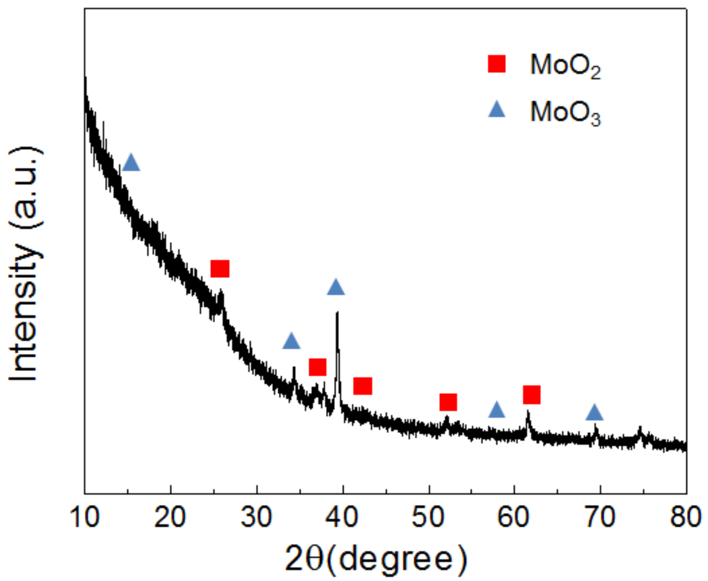

Figure S4

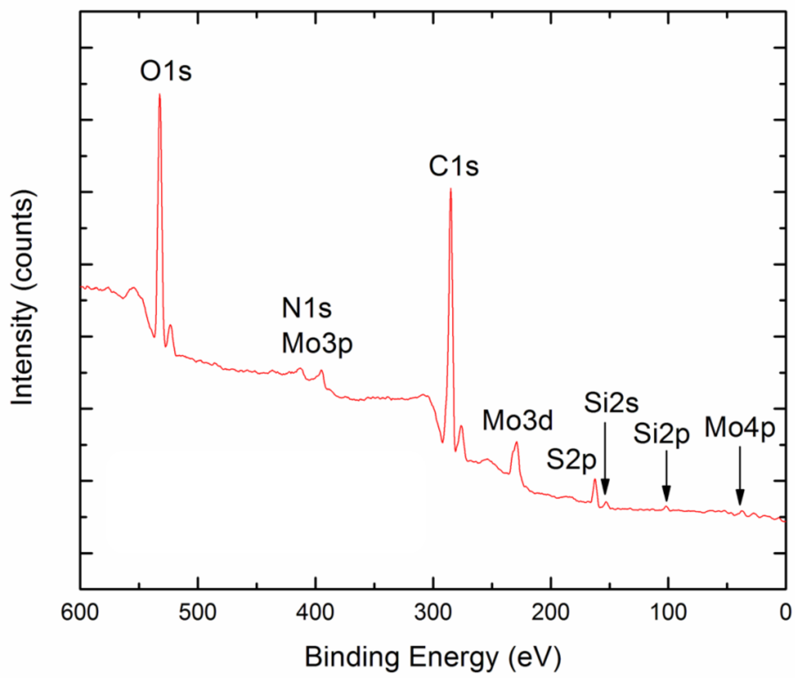
O1s
C1s
N1s
Mo3p
Mo3d
Si2s
S2p
Si2p
Mo4p
Intensity (counts)
600
500
400
300
200
100
0
Binding Energy (eV)

Figure S5

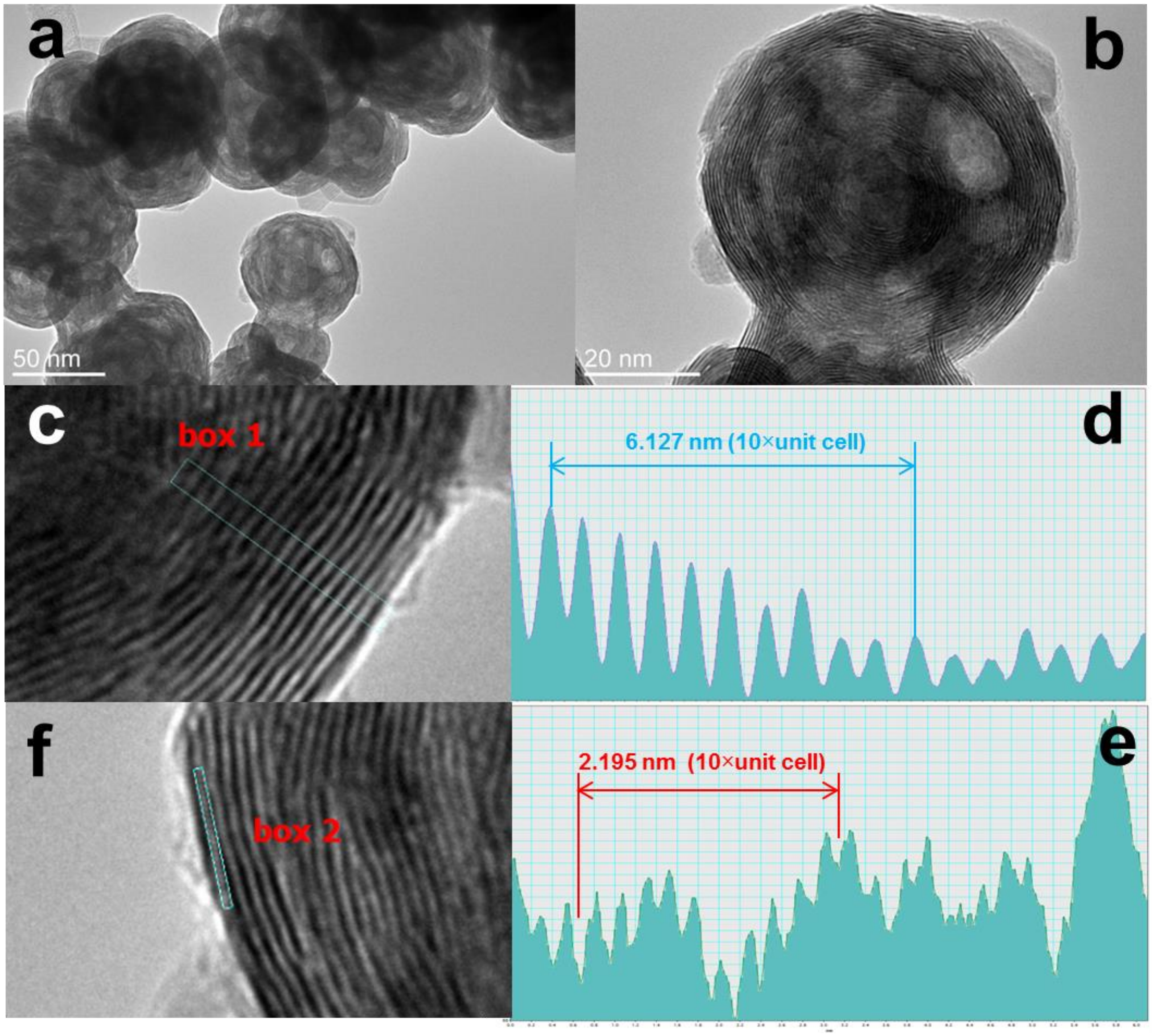
a
50 nm
b
20 nm
c
box 1
d
6.127 nm (10×unit cell)
f
box 2
e
2.195 nm (10×unit cell)

Figure S6

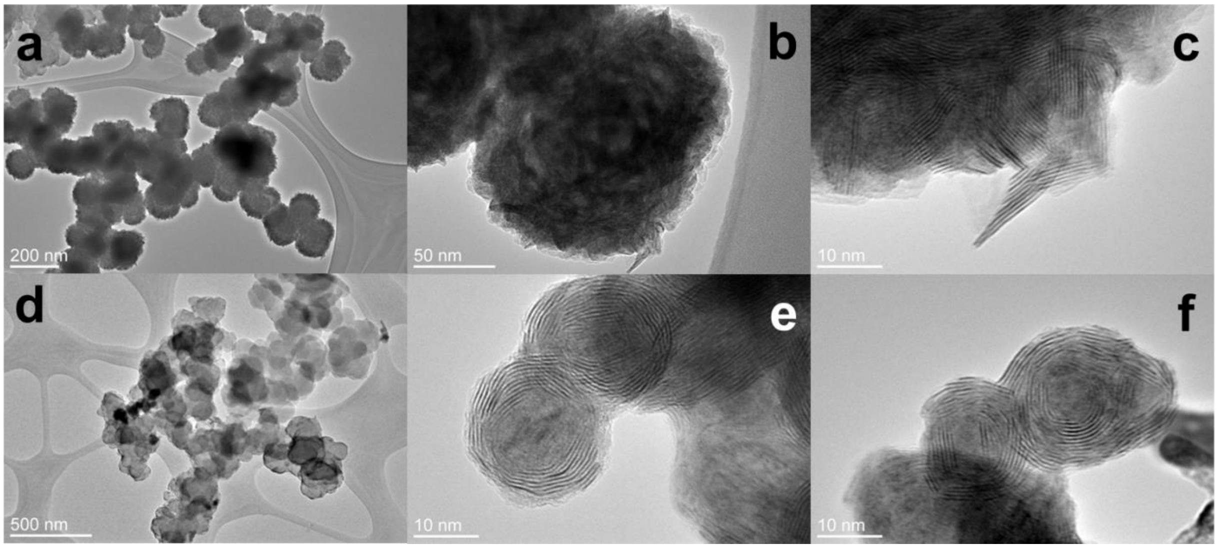

Supplement: Supplementary Information [file srep22503-s1.pdf]
